# Interactions between copy number and expression level of genes involved in fluconazole resistance in *Candida glabrata*

**DOI:** 10.3389/fcimb.2013.00074

**Published:** 2013-11-11

**Authors:** Salma Abbes, Charles Mary, Hayet Sellami, Annie Michel-Nguyen, Ali Ayadi, Stéphane Ranque

**Affiliations:** ^1^Laboratoire de Biologie Moléculaire Parasitaire et Fongique, Faculté de médecine, University of SfaxSfax, Tunisie; ^2^Laboratoire de Parasitologie-Mycologie, Aix-Marseille Université, AP-HMMarseille, France

**Keywords:** *Candida glabrata*, fluconazole, resistance mechanisms, gene expression, gene copy number, quantitative real-time PCR, human infection

## Abstract

**Objectives**: This study aimed to elucidate the relative involvement of drug resistance gene copy number and overexpression in fluconazole resistance in clinical *C. glabrata* isolates using a population-based approach.

**Methods**: Fluconazole resistance levels were quantified using the minimal inhibitory concentration (MIC) via Etest method. Both gene expression levels and gene copy number of *CgCDR1*, *CgPDH1*, *CgERG11*, and *CgSNQ2* were assessed via quantitative real-time PCR. The influence of the main effects and first-level interactions of both the expression level and copy number of these genes on fluconazole resistance levels were analyzed using a multivariate statistical model.

**Results**: Forty-three *C. glabrata* isolates were collected from 30 patients during in a hospital survey. In the multivariate analysis, *C. glabrata* fluconazole MICs were independently increased by *CgSNQ2* overexpression (*p* < 10^−4^) and the interaction between *CgPDH1* gene copy number and *CgPDH1* expression level (*p* = 0.038). In contrast, both *CgPDH1* overexpression (*p* = 0.049) and the interaction between *CgSNQ2* and *CgERG11* expression (*p* = 0.003) led to a significant decrease in fluconazole MICs.

**Conclusion**: Fluconazole resistance in *C. glabrata* involves complex interactions between drug resistance gene expression and/or copy number. The population-based multivariate analysis highlighted the involvement of the *CgSNQ2* gene in fluconazole resistance and the complex effect of the other genes such as *PDH1* for which overexpression was associated with reduced fluconazole resistance levels, while the interaction between *PDH1* overexpression and copy number was associated with increased resistance levels.

## Introduction

*Candida glabrata* is responsible for the increase in cases of systemic and superficial candidiasis in many countries (Diekema et al., 2002; Li et al., 2007; Pfaller and Diekema, 2007). Several reports have revealed that a significant percentage of *C. glabrata* clinical isolates are resistant to fluconazole. Pfaller et al. have found that between 2001 and 2007, isolation frequencies and fluconazole resistance among *C. glabrata* blood stream infection isolates was increased compared with the 1992–2001 period (Pfaller and Diekema, 2004; Pfaller et al., 2009). Similarly, antifungal susceptibility surveys have detected 17 and 18% resistance levels in Europe and North America, respectively (Pfaller et al., 2009). Many studies have shown that drug efflux mediated by the overexpression of ATP-binding cassette (ABC) transporter family membrane proteins was the main molecular mechanism associated with *C. glabrata* fluconazole resistance (Sanglard et al., 1999, 2001; Bouchara et al., 2000; Niimi et al., 2002; Wada et al., 2002; Cernicka and Subik, 2006; Coleman and Mylonakis, 2009). Studies have also shown that among ABC transporters, *CgCDR1*, and *CgPDH1*, but not *CgSNQ2*, play a major role in fluconazole resistance (Sanguinetti et al., 2005; Torelli et al., 2008). Azole resistance in *C. albicans* was primarily associated with mutation or overexpression of ERG11, which encodes the key azole target protein lanosterol 14-alpha demethylase (Chau et al., 2004; Cernicka and Subik, 2006). In contrast, *CgERG11* mutations were marginally involved in *C. glabrata* fluconazole resistance (Sanguinetti et al., 2005). However, *CgERG11* overexpression has been associated with chromosome duplication and aneuploidy due to genome plasticity as well as fluconazole resistance in *Cryptococcus neoformans*, *C. albicans*, and *C. glabrata* (Marichal et al., 1997; Kwon-Chung and Chang, 2012). This study aimed to dissect the relative contribution of drug resistance gene copy number and overexpression on fluconazole resistance in clinical *C. glabrata* isolates.

## Methods

### Patients and isolates

The population of *Candida glabrata* clinical isolates included in the present study were recovered during an epidemiological survey of antifungal resistance conducted at the Habib Bourguiba University Hospital in Sfax from January 2005 to December 2007 as previously described (Abbes et al., 2011, 2012; Amouri et al., 2011; Sellami et al., 2011). The specimens were isolated from 30 patients with urinary tract infection, vaginal infection or systemic infections. *Candida glabrata* was identified by assessing the isolates using Candiselect medium (bioMérieux, Craponne, France) and the ID32C profile (bioMérieux). Isolate fluconazole minimum inhibitory concentrations (MICs) were then measured using the E-test method (AB Biodisk, Sweden) on RPMI 1640 medium (AES, France) as recommended by the manufacturer. Isolates with MICs >64 mg/l were defined as fluconazole resistant (Clinical and Laboratory Standards Institute, 2008).

### RNA and DNA isolation

Total RNA was extracted from yeast cells isolated from RPMI plates during the logarithmic growth phase at 48 h of incubation using an RNAeasy Protect™ mini kit (Qiagen, Courtaboeuf, France) according to the manufacturer's instructions. RNA extracts were treated with RNase-free DNase (Qiagen) to avoid DNA contamination. Genomic DNA was extracted using the NucleoSpin™ Tissue method (Macherey-Nagel, Dueren, Germany) according to the manufacturer's instructions. Genomic DNA (gDNA) extracts were treated with DNase-free RNase (Qiagen) to eliminate RNA contamination.

### Quantitative real-time PCR

Quantitative real-time PCR analysis was performed to measure the expression levels and copy number of each of the four target genes: *CgCDR1*, *CgPDH1*, *CgSNQ2*, and *CgERG11* (Table 1). Gene transcript levels and copy number values were normalized to URA3, a single copy gene encoding orotidine 5-phosphate decarboxylase.

**Table 1:** **Primers and probes used to amplify *CgCDR1*, *CgPDH1* (formerly denoted *CDR2*), *CgSNQ2*, *CgERG11*, and *CgURA3* in separate quantitative Real-Time PCR reactions as described in Wada et al. (2002)**.

**Gene (genbank accession no)**	**Primer or probe**	**Sequence**	**Annealing site (5′3′)**
CgCDR1 (AF109723)	CDR1a	TAGCACATCAACTACACGAACGT	4500–4522
	CDR1b	AGAGTGAACATTAAGGATGCCATG	4647–4670
	CDR1pr	6FAM-TGCTGCTGCTTCTGCCACCTGGTT-TAMRA	4621–4644
CgPDH1 (AF251023)	CDR2a	GTGCTTTATGAAGGCTACCAGATT	164–187
	CDR2b	TCTTAGGACAGAAGTAACCCATCT	251–274
	CDR2pr	6FAM-TACCTTTGCGTGCTGGGCGTCACC-TAMRA	217–240
CgSNQ2 (AF251022)	SNQ2a	ACCATGTGTTCTGAATCAATCAAT	360–383
	SNQ2b	TCGACATCATTACAATACCAGAAA	462–485
	SNQ2pr	6FAM-AACTAATCGCCGCAGGTTGTGACA-TAMRA	394–317
CgERG11 (L40389)	ERGa	ATTGGTGTCTTGATGGGTGGTC	928–949
	ERGb	TCTTCTTGGACATCTGGTCTTTCA	1019–1042
	ERGpr	6FAM-ACTTCCGCTGCTACCTCCGCTTGG-TAMRA	955–978
CgURA3 (L13661)	URAa	GAAAACCAATCTTTGTGCTTCTCT	168–191
	URAb	CATGAGTCTTAAGCAAGCAAATGT	268–291
	URApr	VIC-ACGTCACCACCACCAGCGAATTGT-TAMRA	194–217

#### Reverse transcriptase PCR (RT-PCR)

First, the RNA extract of each isolate was transcribed into cDNA using reverse transcriptase (RT), which was performed with a 25-μl reaction volume containing 2.5 μl MuLva buffer (Biology, France), 2 U of MuLva RT (Biology), 5 μl RNA sample and 0.5 μM random primer and polyA mixture (Eurogentec, France). The samples were subjected to reverse transcription at 37°C for 60 min.

#### Quantitative real-time PCR (qPCR)

Two μl of either cDNA or gDNA template was used to separately assess expression levels and gene copy number of the four genes: *CgCDR1*, *CgPDH1*, *CgSNQ2*, and CgERG11, as previously described (Sanguinetti et al., 2005). The primers utilized are detailed in Table 1. Briefly, 200 pM of each primer, 200 pM hydrolysis probe and 25 μl TaqMan mix (Applied Biosystems, Courtaboeuf, France) were included in the PCR assay. Copy number PCR cycling conditions consisted of an initial step at 94°C for 10 min and then 40 cycles of 15 s at 94°C, 30 s at 55°C and 20 s at 72°C. Fluorescence data were collected during the extension step and analyzed with a MX 4000 real-time PCR system (Stratagene, France). Each reaction was performed in duplicate. Copy number efficiency was determined for each gene. The normalized relative quantities of the gene-specific product or copy number for each sample were then calculated, i.e., the quantity of the samples of interest was compared with a calibrator (TU10, a fluconazole susceptible control, MIC = 0.125 mg/l). The quantity of both the calibrator and the samples of interest were normalized to URA3.

### *C. glabrata* genotyping

The *C. glabrata* isolates were genotyped using six markers, five microsatellites (RPM2, MTI, GLM4, GLM4, and GLM6) and irregular patterns in the ERG3 gene as previously described (Abbes et al., 2011, 2012).

### Statistical analysis

Normalized gene copy numbers and expression levels are indicated using range, median, and interquartile range (IQR). The correlation between fluconazole MICs and the relative expression level and copy number of each gene was analyzed using the Spearman rank correlation coefficient. For qualitative analysis, we selected a threshold value of 2.5 normalized gene expression or normalized gene copy number to define gene overexpression or an increase in gene copy number, respectively. Regarding quantitative analysis, fluconazole MIC values were log-transformed, standardized and centered. The effect on fluconazole MICs due to the gene copy number and expression levels of each of the four genes studied was modeled using the Genmod procedure. All first-level interactions (the product of the two main effect terms) between each covariate were tested. When an interaction was statistically significant, each of the two main effects was retained in the model, regardless of the level of statistical significance. The most parsimonious best-fitting model was selected using the log-likelihood ratio test. After selection of the model, the generalized estimating equation option was used to account for the non-independence of isolates sampled from a same patient. Statistical analyses were performed with SAS 9.2 statistical software (Cary, NC, USA). All statistical tests were two-sided, and *p* < 0.05 was considered significant.

## Results

The characteristics of the 43 *C. glabrata* clinical isolates collected during a hospital survey from 30 patients (9 patients had ≥2 episodes) at the Habib Bourguiba University hospital in Sfax (Tunisia) are detailed in Table 2. Briefly, most isolates were grown from urine samples, and 15 isolates were involved in invasive infections. Fluconazole MICs ranged from 0.016 to 256 mg/l; MIC_50_ and MIC_90_ were 4 mg/l and 256 mg/l, respectively. Nine isolates were classified as resistant to fluconazole with a MIC >256 mg/l. Microsatellite analysis displayed 17 distinct genotypes. Twenty-two isolates were sequentially collected (7–220 days lag in the same patient). In patients 1, 2, 3, and 4 treated with fluconazole, MICs were significantly increased in sequential *C. glabrata* isolates, whereas the microsatellite-based genotypes remained stable (Tables 3, 4). These patients were exposed to 150, 30, 220, or 120 days of fluconazole therapy with a cumulative dose of 4, 5.6, 8, and 12 g fluconazole, respectively (Table 3). In patients 6 and 11, the fluconazole MICs were increased in sequential isolates with a distinct microsatellite genotype, thereby indicating that they probably acquired resistant *C. glabrata* strains upon exposure to fluconazole (Table 2). Finally, both microsatellite genotypes and fluconazole MICs remained stable in the sequential isolates derived from patients 19, 21, and 26 (Table 2).

**Table 2:** **Relative gene expression levels and copy numbers of *CgCDR1*, *CgPDH1*, *CgERG11*, and *CgSNQ2* in fluconazole sensitive and resistant *Candida glabrata* isolates**.

**Patients**	**Isolates**	**Sample site**	**Microsatellite type**	**Fluconazole MIC (μg/ml)**	**Relative increase in**							
					***CDR1 expression***	***CDR1 copies***	***PDH1 expression***	***PDH1 copies***	***ERG11 expression***	***ERG11 copies***	***SNQ2 expression***	***SNQ2 copies***
1	1.1	Urine	3	1.5	3.09	4.59	6.62	1.99	0.4	0.74	1.11	0.18
1	1.2	Urine	3	256	5.64	4.7	20.04	4.13	0.54	1549.94	0.48	364.12
2	2.1	Urine	5	16	ND	2.01	ND	1.85	ND	1.55	ND	2.13
2	2.2	Urine	5	256	92.61	1.83	169.56	1.37	0.12	1.6	5.41	0.94
3	3.1	Vaginal	9	2	0.12	2.16	0.44	1.61	0.24	2.3	0.07	1.58
3	3.2	Vaginal	9	256	0.29	2.18	0.57	1.27	1.16	1.41	0.19	1.65
4	4.1	Urine	1	4	ND	1.48	0.11	1.14	0.53	3.35	0.04	0.99
4	4.2	Urine	1	256	0.1	2.32	9.42	0.83	0.3	3.14	1.19	1.44
5	5	Urine	1	256	1.84	1.11	7.64	1.16	0.56	0.91	0.97	1.22
6	6.1	Urine	8	32	0.1	1.02	0.49	0.9	0.94	0.93	0.56	0.82
6	6.2	Urine	2	256	2.54	4.04	375.57	5.7	4.34	12.7	73.61	0.56
7	7	Blood culture	7	256	0.39	1.89	5.6	2.71	0.42	2.01	5.77	1.2
8	8	Urine	9	256	12.06	3.64	0.31	3.21	0.23	3.74	1.17	3.24
9	9	Blood culture	7	256	12.14	1.27	0.59	0.89	0.87	1.01	2.17	1.03
10	10	Blood culture	7	32	1.42	1.25	0.27	1.01	0.25	1.84	0.26	1.56
11	11.1	Urine	12	1	0.34	ND	15.08	ND	1.11	ND	12.93	ND
11	11.2	Urine	4	32	0.46	1.21	4.86	1.38	0.57	0.79	0.45	1.04
11	11.3	Urine	10	32	0.65	1.25	0.87	0.86	0.54	1.84	1.72	ND
12	12	Blood culture	10	1	0.43	1.41	0.46	2.05	0.09	1.45	0.01	1.28
13	13	Blood culture	6	2	ND	1.73	0.04	1.21	0.27	1.29	0.22	0.85
14	14	Blood culture	10	2	0.3	1.68	0.26	1.17	0.16	1.68	0.37	1.39
15	15	Kidney abscess	13	3	ND	1.59	0.76	1.93	0.42	1.6	0.99	0.7
16	16	Lung abscess	14	2	0.23	1.24	0.35	0.79	0.31	1.07	0.47	0.95
17	17	Urine	14	2	0.18	1.75	0.46	1.52	0.26	3.86	0.42	0.77
18	18	Blood culture	15	2	0.09	1.74	0.25	0	0.26	2.1	0.3	1.12
19	19.1	Urine	7	0.064	0.019	1.26	0.94	1.32	1.23	1.19	0.49	1.31
19	19.2	Urine	7	0.125	0.04	1.2	0.79	1.14	0.38	0.99	0.52	0.69
20	20	Blood culture	7	4	1.3	1.47	17.05	1.67	1.78	1.32	0.48	1.83
21	21.1	Urine	4	6	6.71	1.16	3.59	1.25	2.34	4.03	3.56	0.41
21	21.2	Urine	4	4	0.86	2.76	0.74	2.57	0.71	2.57	0.69	2.82
21	21.3	Urine	4	4	0.82	1.4	0.96	1.51	1.32	1.38	0.79	1.67
21	21.4	Urine	4	8	0.01	2.16	0.57	1.61	1.89	2.3	1.72	1.75
21	21.5	Urine	4	4	2.63	2.31	2.9	1.96	1.05	1.76	2.14	1.6
22	22	Blood culture	3	4	1.59	1.71	1.13	1.75	0.74	1.34	1.18	1.43
23	23	Urine	4	4	1.81	1.62	2.79	1.48	0.52	1.46	1.79	1.61
24	24	Urine	16	4	0.12	1.71	0.29	1.18	0.43	1.1	0.25	1.28
25	25	Urine	10	0.5	0.03	ND	1.61	2.61	0.09	1.36	0.1	48.88
26	26.1	Urine	9	0.016	ND	4.19	2.76	2.23	1	1.15	0.35	0.37
26	26.2	Urine	9	6	0.4	ND	0.88	ND	0.84	ND	0.59	ND
27	27	Blood culture	11	8	ND	ND	0.76	ND	0.7	ND	0.15	ND
28	28	Blood culture	3	1	0.2	4.27	1.16	2.41	2.09	1.59	0.34	0.66
29	29	Blood culture	9	4	ND	1.73	1.26	0.64	0	0.55	0.69	0.77
30	30	Blood culture	17	2	0.01	1.18	1.04	1.09	0.98	0.78	2.08	0.75

**Table 3:** **Characteristics and fluconazole treatment features in six patients infected with *C. glabrata* that acquired *in vitro* resistance under fluconazole treatment**.

**Patient no.**	**Age (yr)/sex**	**Underling disease**	**No. of positive culture (sampling times in days)**	**Fluconazole treatment dosage (administration days)**
1	43/M	Nephrolithiasis	3 (0, 60, 150)	200 mg (60–80)
2	42/F	Diabetes	2 (0, 30)	200 mg (0–28)
3	53/F	Asthma (corticotherapy)	5 (0, 150, 220, 250, 280)	150 mg (120–135), 100 mg (150–165), 400 mg (225–232), 200 mg (250–257)
4	72/F	Nephrolithiasis and diabetes	3 (0, 130, 446)	400 mg (130–160)

**Table 4:** **Details of the relative expression levels and copy numbers of *CDR1*, *PDH1*, *ERG11*, and *SNQ2* genes in four patients' genetically stable *Candida glabrata* sequential isolates that displayed distinct fluconazole MIC levels**.

**Patient**	**Isolate**	**Sampling times in days**	**Fluconazole MIC (mg/l)**	**Microsatellite markers alleles**						**Relative increase in gene**							
				**RPM2**	**MTI**	**ERG3**	**GLM4**	**GLM5**	**GLM6**	***CDR1 expression***	***CDR1 copies***	***PDH1 expression***	***PDH1 copies***	***ERG11 expression***	***ERG11 copies***	***SNQ2 expression***	***SNQ2 copies***
1	1.1	0	1.5	127	238	197	273	298	295	3.09	4.59	6.62	1.99	0.4	0.74	1.11	0.18
	1.2	150	256	127	238	197	273	298	295	5.64	4.70	20.04	4.13	0.54	1549.94	0.48	364.12
2	2.1	0	16	139	227	228	276	262	298	ND	2.01	ND	1.85	ND	1.55	ND	2.13
	2.2	30	256	139	227	228	276	262	298	92.61	1.83	169.56	1.37	0.12	1.6	5.41	0.94
3	3.1	0	2	127	238	197	288	271	295	0.12	2.16	0.44	1.61	0.24	2.3	0.07	1.58
	3.2	60	256	127	238	197	288	271	295	0.29	0.57	0.57	1.27	1.16	1.41	0.19	1.65
4	4.1	0	4	127	238	197	270	271	289	ND	1.48	0.11	1.14	0.53	3.35	0.04	0.99
	4.2	120	256	127	238	197	270	271	289	0.1	2.32	9.42	0.83	0.30	3.14	1.19	1.44

### *CgCDR1*, *CgPDH1*, *CgSNQ2*, and *CgERG11* expression

To investigate the mechanism of fluconazole resistance in *C. glabrata* we analyzed the gene expression levels of *CgCDR1*, *CgPDH1*, *CgERG11*, and *CgSNQ2*. In the 43 *C. glabrata* isolates, the normalized gene expression levels were as follows: *CDR1* levels ranged from 0.010 to 92.61, median 0.415 IQR [0.120–1.825]; *CDR2* levels ranged from 0.04 to 375.57, median 0.91 IQR [0.46–3.59]; *SNQ2* levels ranged from 0.010 to 73.61, median 0.575 IQR [0.34–1.72] and *ERG11* levels ranged from 0 to 4.34, median 0.54 IQR [0.27–1.00] (Table 2).

When considering the nine fluconazole resistant isolates, *CgCDR1* was overexpressed in five isolates, *CgPDH1* was overexpressed in six isolates, *CgSNQ2* was overexpressed in three isolates and *CgERG11* was overexpressed in one isolate (Table 2). Overall, the expression of the efflux pump genes *CgCDR1*, *CgPDH1*, and *CgSNQ2* was increased in two resistant isolates compared with the susceptible initial isolates. One resistant isolate displayed simultaneous overexpression of *CgCDR1*, *CgPDH1* and *CgERG11*, while another isolate displayed overexpression of both *CgPDH1* and *CgSNQ2*. *CgCDR1* and *CgPDH1* were each overexpressed in two resistant isolates. In contrast, at least one gene was overexpressed in nine fluconazole susceptible isolates. Although overexpression of *CgERG11* was never observed, *CgPDH1*, *CgCDR1* and *CgSNQ2* were overexpressed in seven, three and two fluconazole-susceptible isolates, respectively (Table 2). In four of these isolates, *CgPDH1* overexpression was observed in parallel with either *CgSNQ2* or *CgCDR1* overexpression. When considering sequential isolates derived from the same patients, the acquisition of fluconazole resistance was associated with increased expression of *CgCDR1* and *CgPDH1* in patient 1 and increased expression of *CgPDH1* in patient 4 (Table 4).

### *CgCDR1*, *CgPDH1*, *CgSNQ2*, and *CgERG11* copy number

To investigate one possible mechanism of *C. glabrata* fluconazole resistance we analyzed gene copy number of *CgCDR1*, *CgPDH1*, *CgERG11*, and *CgSNQ2*. The normalized gene copy number values were as follows: *CDR1* copy numbers ranged from 1.02 to 4.70, median 1.71 IQR [1.2–2.18]; *CDR2* copy numbers ranged from 0.00 to 5.70, median 1.43 IQR [1.14–1.98]; *SNQ2* copy numbers ranged from 0.18 to 364.12, median 1.22 IQR [0.77–1.61] and *ERG11* copy numbers ranged from 0.55 to 1549.94, median 1.51 IQR [1.13–2.20]. In some isolates, either overexpression or increased gene copy number was observed for *CgCDR1* and *CgPDH1* (Table 2). Nevertheless, three out of five resistant isolates that displayed *CgCDR1* overexpression also displayed an increase in *CgCDR1* gene copy number (4.7, 4.04 and 3.64 copies for isolates 1.2, 6.2, and 8, respectively) (Table 2). Three of the six resistant isolates that displayed *CgPDH1* overexpression also exhibited an increase in *CgPDH1* gene copy number (2.71, 4.13, and 5.7 copies in isolates 7, 1.2, and 6.2, respectively). Similarly, resistant isolates displaying *CgERG11* overexpression also exhibited an increase in *CgERG11* gene copy number (12.7 times more than TU10). For *CgSNQ2*, gene overexpression did not correlate with gene copy number (Table 2). Among the fluconazole susceptible isolates, the copy number of at least one gene was increased in seven isolates. More precisely, copy number of the four genes was increased in isolate 21.2, while only one gene copy number was increased in the remaining isolates as follows: *CgERG11* copy number was increased in four isolates and the gene copy number of *CgSNQ2* and *CgCDR1* was increased, each in one isolate.

### Population-based gene interaction study

**Figure 1:**
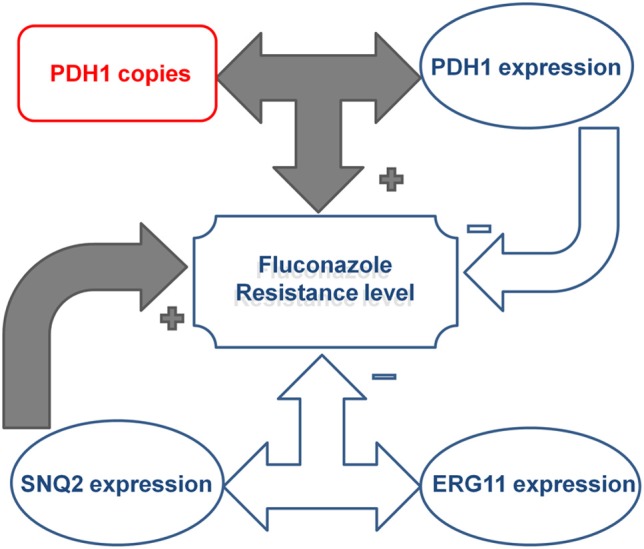
**Illustration of the effect of the complex interactions between *CgPDH1*, *CgSNQ2*, and *CgERG11* drug resistance gene copy numbers and expression levels on fluconazole resistance in *Candida glabrata* clinical isolates**. *CgPDH1* gene copy number and expression were correlated, and their interaction led to an increase in fluconazole MIC levels. In contrast, the main effect of *CgPDH1* overexpression independently led to a decrease in MIC levels. The main effect of *CgSNQ2* overexpression independently promoted an increase in fluconazole MIC levels, while its interaction with *CgERG11* expression led to a decrease in MIC levels.

Univariate correlation analysis indicated a complex interaction between gene copy number and expression levels (Table 5). A correlation between *CgPDH1*, *CgCDR1*, and *CgSNQ2* gene copy numbers was observed, while a correlation between the expression levels of *CgPDH1*, *CgERG11*, and *CgSNQ2* was also found. Fluconazole MIC values positively correlated with both *CgCDR1* and *CgSNQ2* expression levels. A positive correlation between gene copy number and expression level was observed only for the *CgPDH1* gene. Figure 1 summarizes the significant combined main effects and first-level interactions between gene copy number and gene expression levels influencing fluconazole MIC levels in *C. glabrata*.

**Table 5:** **Spearman rank correlation coefficients between the fluconazole MICs and the normalized expression level and copy number of *CDR1*, *PDH1*, *ERG11*, and *SNQ2* genes**.

		**Fluconazole**	**Gene expression**				**Gene copy number**		
		**MIC**	***CDR1***	***PDH1***	***ERG11***	***SNQ2***	***CDR1***	***CDR2***	***ERG11***
**GENE EXPRESSION**									
CDR1		**0.50551**							
PDH1		0.21498	**0.38320**						
ERG11		0.11168	0.04558	**0.34565**					
SNQ2		**0.38022**	**0.41744**	**0.58012**	**0.33921**				
**GENE COPY NUMBER**									
CDR1		0.04854	0.19972	0.18646	−0.04077	−0.00235			
PDH1		−0.04227	0.29146	**0.36136**	0.18184	0.06981	**0.62306**		
ERG11		**0.30065**	0.18173	−0.05340	−0.00061	0.09112	**0.43376**	**0.32972**	
SNQ2		0.29037	0.01279	−0.07420	−0.07782	−0.13971	0.17851	0.24155	0.29510

The Spearman rank correlation coefficient values >0.3 (and p < 0.05) are bolded.

The best-fitting multivariate model of the factors influencing fluconazole MIC values included (i) the main effects of the expression levels of *CgSNQ2*, *CgERG11*, and *CgPDH1* and the gene copy number of *CgPDH1* and (ii) the interaction between *CgSNQ2* and *CgERG11* gene expression and the interaction between *CgPDH1* copy number and expression. In this model, *C. glabrata* fluconazole MICs were significantly increased due to both *CgSNQ2* overexpression (*p* < 10^−4^) and the interaction between *CgPDH1* copy number and expression (*p* = 0.038). In contrast, both *CgPDH1* overexpression (*p* = 0.049) and the interaction between *CgSNQ2* and *CgERG11* expression (*p* = 0.003) led to a statistically significant decreased in fluconazole MICs. Neither *CgERG11* expression (*p* = 0.823) nor *CgPDH1* gene copy number (*p* = 0.985) independently displayed a statistically significant influence on fluconazole MICs. These multivariate analysis findings are illustrated in Figure 1.

## Discussion

This population-based approach allowed us to confirm the finding that ABC transporter family membrane gene overexpression is a pivotal fluconazole resistance mechanism in clinical *C. glabrata* isolates, which had been previously evidenced primarily using laboratory strains. Moreover, this study enabled us to further elucidate the main effects and complex interactions between gene expression and copy number associated with *in vitro* fluconazole resistance. The population-based analysis revealed that *CgPDH1* and *CgERG11* overexpression indirectly influence fluconazole MIC levels by increasing *CDR1* and *SNQ2* expression levels. Although *CgERG11*, *CgSNQ2*, and *CgPDH1* expression and, to a lesser extent, copy number levels were significantly associated with fluconazole resistance, our findings highlight the complex interaction between these genes and suggest the involvement of unmeasured confounding effects. Indeed, mutations in *CgPDR1*, *CgERG11* or others genes that might impact *C. glabrata* fluconazole resistance were not analyzed in this study. Univariate analysis (Table 5) suggested that *CgCDR1* overexpression plays a major role in the regulation of MIC levels. However, its effect did not remain significant in the multivariate analysis, which highlighted the major independent effect of *CgSNQ2* overexpression. Both univariate and multivariate analyses showed that copy number and overexpression were markedly correlated for *CgPDH1* but not for the other genes. In patient 1, for whom sequential *C. glabrata* isolates displayed a stable genotype, switching to fluconazole resistance was associated with both overexpression and increased gene copy number of *CgPDH1* (Table 4). A high correlation between the expression levels of all membrane transporter genes was detected, which is probably because they are controlled by the same transcription factor *CgPDR1* (Ferrari et al., 2009; Paul et al., 2011). This transcription factor has been associated with both resistance to azole antifungals via the overexpression of ABC transporter genes and enhanced virulence in *C. glabrata* due to gain-of-function mutations (Ferrari et al., 2009).

*CgCDR1* and *CgPDH1* overexpression-driven drug efflux is the most frequent fluconazole resistance mechanism observed in *C. glabrata* (Miyazaki et al., 1998; Sanglard et al., 1999, 2001; Wada et al., 2002; Coleman and Mylonakis, 2009). Numerous reports have documented acquired resistance upon exposure to long-term fluconazole therapy (Coste et al., 2007; Shin et al., 2007), as observed in the four patients who acquired resistant isolates (Table 3). Although *CgCDR1* and *CgPDH1* expression could not be determined in the fluconazole susceptible isolate, overexpression of both genes was associated with fluconazole resistance in the isolate derived from patient 2. Acquisition of both *CgPDH1* overexpression and increased gene copy number was observed in isolates derived from patient 1 (Table 4).

An overproduction of lanosterol-demethylase in *C. glabrata* has initially been described by Marichal et al. who found that duplication of the entire chromosome E (Marichal et al., 1997), the chromosome on which *ERG11* is located, was associated with a four-fold increase in *ERG11* copy number in fluconazole resistant compared with susceptible isolates. A recent study has also shown that lanosterol-demethylase overproduction, for which the mechanism is unknown, was involved in fluconazole resistance (Redding et al., 2003). In our study, an increase in *CgERG11* gene copy number was associated with *CgERG11* overexpression in only one isolate. Furthermore, *CgCDR1*, *CgPDH1*, and/or *CgERG11* gene copy number was inconsistently associated with overexpression, thereby suggesting that an increase in gene copy number might sometime result in gene silencing via either heterochromatin formation or DNA methylation (Merrick and Duraisingh, 2006; Mishra et al., 2011).

A strong correlation between *CgCDR1*, *CgPDH1*, and *CgERG11* gene copy number was observed (Table 4), although these genes are located on chromosomes M, F, and E, respectively. Further genome hybridization experiments may shed light on the underlying mechanisms and enable the localization of these amplified genes. Many gene duplication mechanisms have been described in various regions of the *C. glabrata* genome. Poláková et al. have suggested that duplication occurs in chromosomal segments that encode for some ABC transporter genes (Poláková et al., 2009). Similarly, Mûller et al. have shown that the mechanisms of microevolution of this asexual species include recombination within tandem arrays of repeated genes, which often encode cell wall proteins, thereby suggesting a possible adaptive function (Mûller et al., 2009). The fact that some genotypically identical isolates (genotype 3 and 7, Table 2) displayed different fluconazole resistance mechanisms support the hypothesis that fluconazole resistance is chiefly associated with the overexpression of normally present genes within the C. glabrata genome rather than gene mutation.

In conclusion, this population-based approach provides further evidence that overexpression of membrane transporter genes is a pivotal fluconazole resistance mechanism in *C. glabrata* clinical isolates. A high correlation between the expressions of these genes, which are controlled by the same transcription factor, was observed. However, the multivariate analysis revealed, for the first time, that *CgSNQ2* exerts a major independent effect on fluconazole resistance. Regarding the other genes assessed, interactions played a greater role than the main effects in fluconazole resistance. Notably, *CgPDH1* overexpression was significantly associated with increased gene copy number, which was not observed with the other genes analyzed. Furthermore, a correlation between gene copy number and fluconazole resistance was only observed for *CgERG11*, thereby suggesting that an increase in copy number of *C. glabrata* membrane transporter genes might result in gene silencing. This study provides only partial insight into *C. glabrata* fluconazole resistance mechanisms, as many other mechanisms have not been analyzed, including those targeting other membrane components. Nevertheless, our findings highlight the important role that complex gene copy number and overexpression interactions play in fluconazole resistance, which should been taken into account in further studies.

## Author contributions

Salma Abbes carried out the collection of the clinical isolates, the molecular genetic studies and drafted the manuscript. Charles Mary participated in the design of the molecular genetic studies and drafted the manuscript. Hayet Sellami participated in the study design and isolates and clinical data collection. Annie Michel-Nguyen participated in *C. glabrata* isolates' identification and *in vitro* susceptibility assays analysis. Stéphane Ranque performed the statistical analysis. Ali Ayadi and Stéphane Ranque conceived the study, and participated in its design and coordination and helped to draft the manuscript. All authors read and approved the final manuscript.

### Conflict of interest statement

The authors declare that the research was conducted in the absence of any commercial or financial relationships that could be construed as a potential conflict of interest.
